# Motor Preparation for Action Inhibition: A Review of Single Pulse TMS Studies Using the Go/NoGo Paradigm

**DOI:** 10.3389/fpsyg.2019.00340

**Published:** 2019-02-21

**Authors:** Stefania C. Ficarella, Lorella Battelli

**Affiliations:** ^1^ Center for Mind/Brain Sciences, University of Trento, Rovereto, Italy; ^2^ Center for Neuroscience and Cognitive Systems@UniTn, Istituto Italiano di Tecnologia, Rovereto, Italy; ^3^ INSERM U 1127, Institut du Cerveau et de la Moelle épinière, Paris, France; ^4^ Berenson-Allen Center for Noninvasive Brain Stimulation and Department of Neurology, Beth Israel Deaconess Medical Center, Harvard Medical School, Boston, MA, United States

**Keywords:** Go/NoGo, action inhibition, TMS, MEP, single pulse, CSE

## Abstract

Human behavior must be flexible to respond to environmental and social demands, and to achieve these goals, it requires control. For instance, inhibitory control is used to refrain from executing unwanted or anticipated responses to environmental stimuli. When inhibitory mechanisms are inefficient due to some pathological conditions, such as attention-deficit hyperactivity disorder (ADHD) or pathological gambling, patients show a reduced capability of refraining from executing actions. When planning to execute an action, various inhibitory control mechanisms are activated to prevent the unwanted release of impulses and to ensure that the correct response is produced. A great body of research has used various cognitive tasks to isolate one or more components of inhibitory control (e.g., response selectivity) and to investigate their neuronal underpinnings. However, inter-individual differences in behavior are rarely properly considered, although they often represent a considerable source of noise in the data. In the present review, we will address this issue using the specific case of action inhibition, presenting the results of studies that coupled the so-called Go/NoGo paradigm with non-invasive brain stimulation to directly test the effects of motor inhibition on the excitability of the corticospinal system (CSE). Motor preparation is rarely measured in action inhibition studies, and participants’ compliancy to the task’s requests is often assumed rather than tested. Single pulse transcranial magnetic stimulation (TMS) is a powerful tool to directly measure CSE, whose responsivity depends on both excitatory and inhibitory processes. However, when motor preparation is not measured and the task design does not require participants to prepare responses in advance, fluctuations in CSE levels can be mistaken for active inhibition. One way to isolate motor preparation is to use a carefully designed task that allows to control for excessive variability in the timing of activation of inhibitory control mechanisms. Here, we review single pulse TMS studies that have used variants of the Go/NoGo task to investigate inhibitory control functions in healthy participants. We will identify the specific strategies that likely induced motor preparation in participants, and their results will be compared to current theories of action inhibition.

## Introduction

To investigate action inhibition in the laboratory, researchers use cognitive tasks that require participants to either execute or inhibit a motor response. The Stop Signal (SST, Logan and Cowan, 1984) and the Go/NoGo (GNG; Donders, 1868/1969) tasks, typically used in action inhibition paradigms, involve overlapping but distinct response inhibition brain circuits (Swick et al., 2011). Despite the differences, neuroimaging studies compare Go with Stop/NoGo conditions to identify the neuronal networks involved in inhibitory control.

A long line of research using different paradigms in which action inhibition is explicitly (SST and GNG) or implicitly (choice or delayed RT tasks) required has suggested that several inhibitory mechanisms may co-occur during response preparation. Preparing an action entails a combination of several cognitive control mechanisms (Wong et al., 2015) that interact with the cognitive context within which such actions are prepared (Vidal et al., 2018). For instance, impulse control is thought to reduce the excitability of selected effectors to prevent the unwanted execution of anticipated responses (Touge et al., 1998; Duque and Ivry, 2009; Duque et al., 2010; Aron, 2011), and a typical example is holding the impulse to press the car’s accelerator pedal, while the traffic light is still red. A suppression of task-irrelevant inputs might be necessary to efficiently and selectively prepare the goal-directed movement (Hasbroucq et al., 1997, 1999). Even quasi-automatic movements induced by moving targets activate fast inhibitory processes for motor preparation (Lara et al., 2018). Action selection (Burle et al., 2004) and competition resolution (Duque and Ivry, 2009; Duque et al., 2010) mechanisms, instead, ensure that the appropriate action is executed. Finally, reactive inhibition allows the quick interruption of responses, upon the presentation of a Stop or NoGo stimulus, such as aborting the action to press the accelerator pedal when a pedestrian suddenly crosses the street (Aron, 2011). Depending on the adopted task, one or more of these inhibitory control functions may be activated at different moments in time and interact with the subthreshold increase of activity in primary and secondary motor areas. While a detailed description of the inhibitory processes activated during motor preparation is beyond the scope of this review (see Duque et al., 2017), here we selected action inhibition studies employing the Go/NoGo paradigm to point out the importance of taking into account motor preparation when investigating action inhibition.

Generally, in the Go/NoGo task, participants are presented, on each trial, with either a stimulus requiring the execution of a predefined response (Go signal) or a different stimulus to which participants are asked not to respond (NoGo signal). Most studies assume that, on trials requiring response inhibition, a previously planned motor response will be interrupted (SST) or withheld (GNG). However, while in the SST the stop signal occurs after a variable delay following the Go stimulus, closer to the time of action execution, there is no guarantee, and little or no empirical evidence, that on trials of the GNG task participants will prepare the action at each trial onset.

Despite the extensive use of the GNG task to localize the brain areas involved in action inhibition, motor preparation at trial onset is, in fact, often assumed rather than tested. In a recent study, Wessel (2017) quantified, through electroencephalographic (EEG) measures of motor preparation, the variability of required inhibitory control functions to prevent the execution of *prepotent* responses in GNG tasks. Variability of inhibitory functions was derived from the probability of NoGo trials and the allotted time to respond, in line with Casey et al. (1997). Wessel’s study concluded that active inhibition mechanisms, as indexed by the P3 component in event-related potentials (ERPs), are less active (up to 75%) on slow-paced GNG tasks with equiprobable Go and NoGo stimuli. Later, a functional magnetic resonance imaging (fMRI) study comparing GNG tasks with rare (25%) vs. prevalent (75%) NoGo trials found functional evidence for the involvement of parietal (rather than frontal like in previous studies, see Swick et al., 2011 for a review) areas in the inhibition of *prepotent* responses, when responses are prepared in advance (Kolodny et al., 2017). Furthermore, physiological evidence suggests that individual differences in corticospinal excitability are linked to differences in response speed (reaction times, RTs) and the concentration of the inhibitory neurotransmitter GABA in the primary motor cortex (Greenhouse et al., 2017). Hence, even if GNG tasks may explicitly ask participants to prepare the response before the onset of the imperative stimulus (IS, Go or NoGo), participants’ responses will still be highly variable. If one assumes that motor preparation occurs on every trial for every participant, without directly testing it, high level of noise might be introduced in the data, thus reducing test-retest reliability.

Some of the strategies adopted by researchers to motivate participants to prepare the response on each trial include cueing the response, manipulating the ratio between Go and NoGo trials, limiting the time to respond, and adopting reward-predicting stimuli. While cues can motivate participants to “get ready” to respond, their effect on physiological measures of response readiness is greatly dependent on the time interval between the cue and the target onset (foreperiod, see Hasbroucq et al., 1997, 1999; Touge et al., 1998; Lebon et al., 2015). Generally, shorter foreperiods (500 ms) induce stronger motor preparation than long ones (2000 ms or longer) (Davranche et al., 2007; Tandonnet et al., 2010).

Single pulse TMS, a non-invasive brain stimulation procedure, can be used to elicit motor evoked potentials (MEPs), a direct measure of corticospinal excitability (CSE), with an excellent temporal resolution. A TMS coil is placed on the scalp of the participants, and it delivers a strong magnetic pulse that reaches the underlying cortical structures virtually unmodified. The magnetic field induces an electrical current in the neuronal populations depending on their axonal orientation, and when applied to the primary motor cortex (M1) at appropriate intensities, it can generate MEPs, which are recorded with surface electromyographic (EMG) electrodes. MEPs reflect the activation of both spinal and cortical neurons (Hallett, 2000), dependent on both inhibitory and excitatory inputs to M1 (Rothwell et al., 1991; Reis et al., 2008). CSE measures and their variability (Klein-Flügge et al., 2013) can be used to track motor preparation (Rossini et al., 1988; Bestmann et al., 2008; Bestmann and Duque, 2016) and inhibition (Stinear et al., 2009). However, the time course and the muscle selectivity of this effect greatly depend on the task, the duration of the foreperiod during which planned responses have to be withheld (Hasbroucq et al., 1997, 1999; Tandonnet et al., 2010; Kennefick et al., 2014; Lebon et al., 2015), and on individual resting excitability levels (Greenhouse et al., 2017). Therefore, ongoing motor preparation is not necessarily signaled by an increase in MEP amplitude (Klein-Flügge et al., 2013), as different inhibitory processes may co-occur (Duque et al., 2017). Finally, proactive inhibitory mechanisms may interact with reactive ones making the data difficult to interpret (Lavallee et al., 2014). As a result, motor preparation should always be assessed when investigating action inhibition using GNG paradigms.

In this review, we aim at summarizing and comparing, whenever possible, the main results of action inhibition studies, in which healthy adults performed GNG tasks (requiring a motor response on Go trials), while recording TMS-elicited MEPs. While an extensive literature review on action inhibition studies can be found elsewhere (Duque et al., 2017), we purposefully kept the inclusion criteria restricted to studies that *only* analyzed single pulse TMS paradigms with a Go/NoGo procedure that required subjects to execute a *manual* response on Go trials. For instance, some Go/NoGo studies require participants to contract the muscles already at baseline (e.g., Begum et al., 2005; Nakata et al., 2006; Kinoshita et al., 2007), and these studies are not reviewed here because the MEPs recorded during high force levels can be due to the activation of spinal motor neurons to a greater extent (Kaneko et al., 1996; Muellbacher et al., 2000). We did, however, review the results of Kinoshita et al. (2007) and Hannah et al. (2018) since the authors asked their participants to weakly contract the muscle at baseline, measuring MEPs that predominantly reflect M1 excitability (Di Lazzaro et al., 1998).

A summary of the methods adopted in each study to measure MEPs is reported in Table 1. We will underline the strategies that previous studies have adopted to induce motor preparation, with the objective to suggest how future studies should consider all important variables and pitfalls when investigating action inhibition. In particular, we will examine the existing literature on motor preparation and action inhibition often employing delayed simple or choice RT tasks to elucidate the differential role of action selection, competition between alternative responses, and the effect of the foreperiod duration on corticospinal excitability.

**Table 1:** Summary of brain stimulation procedures used for the GNG paradigms of reviewed studies.

Study	TMS intensity	Muscle(s)	Pulse time	MEPs normalization
Hoshiyama et al., 1996	115% rMT	Radial extensor, ulnar flexor	IS+150 ms	Neglect (control) condition
Hoshiyama et al., 1997	Between 50 and 60% mso to induce MEPs of 0.5 mV in wrist extensor/flexor muscles	Wrist extensor/flexor, thenar	Randomly in 0-300 ms post-IS window	Neglect (control) condition
Leocani et al., 2000	102–105% rMT	EPBs	Randomly in 20-400 ms post-IS window	Rest
Waldvogel et al., 2000	10% mso above rMT	flexor digitorum communis	200, 300, 400, or 500 ms post-IS	Before and after task instructions (rest?)
Yamanaka et al., 2002	120% rMT	FDI	15 time points from 20 to 300 ms post-IS	−100, −50, and 0 ms relative to IS onset
Kinoshita et al., 2007	Inducing MEPs of 1–1.5 mV in the precontracted muscle (5% mvc)	FDI	IS -500 ms	Precontracted muscle trials without IS
Fujiyama et al., 2011	120% rMT	FPB	WS and IS onset, PreMT	Rest
Fujiyama et al., 2012	120% rMT	FPB	WS + 250 ms, IS onset, ¼, ½, and ¾ of PreMT	WS onset
Freeman et al., 2014	Inducing MEPs half of the Max MEP recorded during practice session	FDI, ADM	IS+250 ms	IS onset on ‘Null’ (catch) trials
Freeman and Aron, 2016	Inducing MEPs half of the Max MEP recorded during practice session (44.7 ± 8.16% mso)	FDI	100, 150, 200, and 250 ms post-IS	IS onset -500 ms
Saumur and Mochizuki, 2018	110% rMT	Right tibialis anterior	IS-1000 ms	Rest
Hannah et al., 2018	Inducing a mean MEP amplitude of 1 mV during 5–10% mvc	FDI	WS, IS, 35% or 70% RT for Go trials; 35% or 70% RT for NoGo trials	WS expected time for IS-TMS MEPs, IS onset for late MEPs

mV, millivolt; rMT, resting motor threshold; mso, maximal stimulator output; EPB, extensor pollicis brevis; FDI, first dorsal interosseous; FPB, flexor pollicis brevis; ADM, abductor digiti minimi; ms, milliseconds; WS, warning signal; IS, imperative stimulus (Go or NoGo); PreMT, premotor time; mvc, maximal voluntary contraction.

## Motor Preparation and Action Inhibition in Single Pulse TMS Studies using the GNG task

In the study of Hoshiyama et al. (1996), participants performed a Go/NoGo task, in which the Go and NoGo conditions were compared to a “neglect” condition, in which subjects were asked not to respond, keeping the muscles relaxed. Each condition was associated with the presentation of a specific order of four colored circles (red or blue). Interestingly, while Go and NoGo trials only differed in the last stimulus presented (blue-blue-blue-**blue** vs. blue-blue-blue-**red**, respectively), the control neglect condition could be already identified from the first presented stimulus (red).

The authors compared MEPs’ amplitudes from the task-agonist (radial extensor) and antagonist (ulnar flexor) muscles recorded at 150 ms after the presentation of the last stimulus between the neglect condition and both the Go and the NoGo conditions. In their paradigm, the neglect condition can be identified from the first, fully informative, stimulus; however, the authors measured MEPs 150 ms after the presentation of the *last* stimulus when, most likely, after a potential initial inhibition, the agonist muscle was back at baseline rest. Nonetheless, the authors reported suppression of MEPs in both the agonist and antagonist muscles on NoGo trials, compared to the control condition. The authors interpreted this suppression as evidence of global motor suppression, presumably engaged when fast reactive interruption of responses is required (Aron and Verbruggen, 2008). Moreover, the authors found that, compared to the neglect condition, Go trials elicited a significant increase and decrease of MEP amplitudes in the task-relevant and task-irrelevant muscle, respectively, in line with inhibition for competition resolution (Duque and Ivry, 2009; Duque et al., 2010) mechanisms involved in action selection processes (Burle et al., 2004).

In a later study (Hoshiyama et al., 1997), the authors investigated the time course of the reported MEP suppression using the same paradigm, while recording MEPs at random intervals within the first 300 ms following the presentation of the last stimulus. MEPs were recorded from agonist (wrist flexor), antagonist (wrist extensor), and the thenar muscle and normalized relative to MEPs recorded in the neglect condition. The authors replicated the results of Hoshiyama et al. (1996) for Go trials, while they found significant MEPs suppression in all three muscles on NoGo trials in the 100–250 ms post-(last) stimulus temporal window. In this paradigm, the Go condition is presented on one third of the trials, a condition that would make one question the reliability of the net inhibition resulting from the comparison to the NoGo-neglect trials (Wessel, 2017). As in Hoshiyama et al. (1996), since MEPs were only recorded after the presentation of the last stimulus, it is impossible to determine whether there was a build-up of motor preparation on Go/NoGo trials and, consequently, how to interpret the MEPs recorded in the neglect condition. One possibility is that, following the first, fully informative, stimulus of the neglect condition, the agonist muscle underwent automatic activation, immediately suppressed by inhibitory mechanisms linked to impulse control. By the time MEPs are recorded at the presentation of the last stimulus, the wrist flexor likely went back to resting levels. If the MEPs’ amplitudes reported in the neglect condition represent resting levels, then global action inhibition could, indeed, be taking place on NoGo trials (and selective inhibition for action selection on Go trials). However, the effect of the presentation of visual stimuli following the first red circle on task-relevant radial extensor and irrelevant ulnar flexor muscles is hard to predict. This is because the neglect condition could be misleading, in that the significant difference in MEPs’ amplitudes recorded from the radial extensor muscle between Go and NoGo trials could be ascribed to motor facilitation and not to an active inhibitory component. The same comparison performed on the ulnar flexor muscle did not yield significant results. Taken together, the results presented by Hoshiyama and colleagues are not conclusive (Hoshiyama et al., 1996, 1997), although they provide the first evidence of possible inhibitory mechanisms for action selection using the GNG task.

In a different study, Leocani et al. (2000) investigated CSE levels comparing different RT tasks in the same group of participants. They adopted an acoustic Go/NoGo task in which participants were instructed to extend their right thumb if a specific tone was presented (on half of the trials) and not to move otherwise. They measured MEPs from the *extensor pollicis brevis* (EPB) of both sides at random post-stimulus delays between 20 and 400 ms and normalized them with resting MEPs. On Go trials, they reported no difference in the amplitude of MEPs, compared to resting levels, in the temporal window of 400-140 ms premovement onset. Starting from 100 to 120 ms before movement initiation, the authors found a significant facilitation in the right EPB and no significant inhibition in the left EPB. Similar results were found in a separate block in which participants had to respond to the go stimulus using the left hand. The lack of difference in MEPs’ amplitude between the two muscles and rest levels up to 120 ms before movement onset suggests that participants did not prepare the motor response in advance. On NoGo trials, the authors reported bilateral facilitation in the temporal window 20-100 ms after the tone with a delayed, bilateral inhibition (between 260 and 300 ms post-stimulus, depending on the block). In this study, even if participants performed a unilateral thumb extension (either right or left depending on the block), they did not prepare the response in advance, since MEPs recorded from both EPBs did not differ from resting levels on Go trials up to 120 ms before movement onset. Moreover, both muscles were at resting levels or higher on NoGo trials up to 100 ms post-stimulus, suggesting that, after an initial unspecific activation of both muscles, the NoGo stimulus triggered global reactive inhibition, similar to what Hoshiyama and colleagues previously found (Hoshiyama et al., 1996, 1997). Similarly, Go and NoGo stimuli in this study elicited bilateral motor preparation and response selection occurred afterward, indexed by selective inhibition on Go trials and global inhibition on NoGo trials. Therefore, we conclude that the results of this study do not represent an example of inhibition of a prepotent response. While an inhibitory process was most likely activated on NoGo trials after an initial muscle-aspecific activation, it was delayed after the preparation process.

Waldvogel et al. (2000) measured MEPs recorded from the *flexor digitorum communis* at different time points between 200 and 500 ms following equiprobable Go and NoGo visual stimuli. Baseline MEPs were recorded before and after the task instruction, although the authors did not specify whether participants were at rest. The authors found reduced MEPs, compared to baseline, on NoGo trials at all time points. Six out of eight subjects that reported NoGo-induced MEP suppression also participated in an fMRI study, which showed an increase in M1 activity following Go stimuli and no significant change from baseline on NoGo trials. Despite the low temporal resolution of the neuroimaging technique, this evidence suggests that, as in previous studies, participants only prepared the action upon stimulus onset and, eventually, inhibited it on NoGo trials. While an activation-inhibition pattern similar to the one reported by Leocani et al. (2000) could be present on the NoGo trials of this study, the earliest TMS pulse delivered by Waldvogel et al. (2000) was 200 ms post-IS. One might hypothesize that had the authors measured MEPs at earlier time points, they would have likely found MEP enhancement on both Go and NoGo trials.

Yamanaka et al. (2002) compared two conditions of the GNG task with opposite motor commands. In one condition (PushGo), participants were asked to press a button with the index finger on Go trials and not to respond after a NoGo stimulus, while in another condition they kept the button pressed at baseline and either release it on Go (ReleaseGo) or keep pressing it on NoGo trials. Single TMS pulses were delivered at 18 different post-stimulus time points, and the recorded MEPs (from the *flexor digiti minimi – FDI –* muscle) were normalized using baseline CSE values (TMS pulses given at −100, −50, or 0 ms relative to stimulus onset). Baseline MEPs did not differ between Go and NoGo trials for both task conditions. In the ReleaseGo condition, participants executed an action (button press) already at baseline, and the intensity of the executed action was measured with continuous electromyographic (EMG) recordings. Any evidence of action inhibition taking place on Go trials can, therefore, be linked to the motor suppression of an ongoing action rather than inhibition of a motor plan. Conversely, while inhibition of a planned response could, in principle, be involved in the NoGo trials of the PushGo condition, the authors did not provide evidence of prior motor preparation, since resting MEPs were not recorded.

As expected, relative to baseline levels, post-stimulus MEPs recorded on NoGo trials were significantly inhibited for the PushGo condition, in which participants should not respond, and significantly enhanced in the ReleaseGo condition, whose NoGo trials require participants to keep pressing the button. Both changes occurred starting from 160 ms post-stimulus onset. Similarly, starting from 160 ms after the presentation of the Go stimulus, the authors found significant MEPs suppression in the ReleaseGo condition. While in the ReleaseGo condition an action was already occurring at baseline, the authors also found MEP suppression on NoGo trials of the PushGo condition, despite being Go and NoGo stimuli occurring on 50% of the trials. Interestingly, in this paradigm, a non-informative warning signal (a beep) was presented 1.8–2.2 s before targets. As initially mentioned, cueing the response in advance can motivate participants to “get ready” to respond (Jaffard et al., 2007). While the presentation of the warning signal could have induced motor preparation at baseline also in the PushGo condition, the absence of MEPs recorded within the foreperiod and at rest limits the interpretation of these data.

Kinoshita et al. (2007) recorded MEP amplitudes in the FDI muscle during the foreperiod (TMS pulse delivered 1.5 s after WS, during a 2-s long foreperiod) of both GNG and simple RT tasks. In both tasks, participants were asked to maintain isometric abduction force in the index finger at 5% of maximum voluntary contraction (MVC), following the presentation of the WS (a 600-ms long auditory tone). Data were normalized using trials where participants held the 5% MVC, without response signals. The authors reported reduced MEP amplitudes during the foreperiod for both the simple RT and GNG tasks.

More recently, Fujiyama et al. (2011) adopted a cued GNG task with a short foreperiod (500 ms) to boost participants’ motor preparation on each trial, despite using equiprobable stimuli. A 500-ms long WS (orange light emitting diode – LED) was immediately followed by an imperative stimulus (IS), either green (Go trials) or red (NoGo trials) LED for 500 ms. In this task, participants were explicitly instructed to prepare the response, a right thumb button press, during the WS interval and to execute it as quickly as possible (within 1,000 ms) upon the presentation of the green LED. MEPs were recorded from the *flexor pollicis brevis* (FPB) muscle. Single pulse TMS-elicited MEPs were recorded at rest. During the task, TMS pulses were delivered at one of three possible time points: at WS onset, IS onset (Go or NoGo), and at a previously calculated average time of EMG activity onset (premotor time, PreMT). The authors expected motor preparation, supposedly triggered by WS onset, to be maximal at IS onset. The PreMT is thought to reflect the duration of central processes for motor preparation (Hasbroucq et al., 1995). A comparison of Go and NoGo trials yielded significant results for the last time point only (PreMT), consistent with previously found enhanced CSE levels before the onset of voluntary movements. Moreover, within-condition comparisons showed that, while MEPs remained stable across the three time points in the NoGo condition, MEPs at PreMT were significantly higher than earlier time points, for Go trials. If motor preparation occurred on each trial following WS onset, one would expect CSE levels to be different than baseline after WS onset and to be further inhibited on NoGo trials, following IS onset. MEP amplitudes were instead unchanged within the course of NoGo trials, which argues against the presence of task-triggered inhibitory mechanisms. Finally, since the authors did not explicitly compare MEPs at rest with MEPs recorded from the GNG task, it is hard to demonstrate that motor preparation occurred at all. Despite the authors’ effort to motivate participants to consistently prepare the response on each trial, the equiprobable Go and NoGo stimuli, coupled with a relatively long allotted time to respond (1,000 ms), might have made participants less compliant to the task requests. Results indeed only showed evidence of motor preparation on Go trials, upon presentation of the Go stimulus, without any evidence of action inhibition on NoGo trials.

To better investigate the time course of CSE during the motor preparation phase, Fujiyama et al. (2012) used a similar paradigm, using a rare NoGo stimulus presentation (30%) to motivate participants to prepare the response at each trial onset. Again, MEPs were recorded from the FPB muscle and resting MEPs were also recorded for comparison. During the task, CSE was assessed at five different time points: at WS onset, 250 ms following the WS onset (during the foreperiod), and at three different intervals following IS onset. Specifically, as in the previous study, they calculated the baseline PreMT and delivered TMS at ¼, ½, and ¾ of the average PreMT. Catch trials, in which the IS was not presented, were added to prevent premature responses and keep participants attentive to the task. During the task, participants were asked to execute thumb flexions in response to Go stimuli. The authors tested a group of younger (*N* = 13, mean age = 26 years) and older (*N* = 13, mean age = 65.5 years) participants. We report results from the two groups separately, since their differences are relevant to the current review. The authors tested whether during the task participants’ MEPs were significantly enhanced, compared to resting MEPs, and found significant differences in both groups. Specifically, MEPs in both trial types were significantly greater than resting levels at all time points they tested, whereas MEP amplitudes in the elderly group was significantly enhanced only at the ¾ PreMT time point of Go trials. In this study, the authors demonstrated that manipulating NoGo stimulus probability induced the younger participants to prepare the response at each trial onset. Therefore, only MEPs from this group will be further commented. MEPs recorded during the task were normalized using MEPs recorded at WS onset and compared across trial type and time points. Results showed, as expected, an increase in MEPs amplitude in the Go, compared to the NoGo condition, following the IS onset, specifically at ½ and ¾ PreMT. In both conditions, MEPs were significantly suppressed, compared to the other time points, at the first time-point following IS onset (¼ of PreMT). A long line of evidence has supported the idea that CSE decrease is a marker of response readiness when a relatively short foreperiod (500 ms) is used (Hasbroucq et al., 1997, 1999; Davranche et al., 2007). The condition-aspecific MEPs suppression found in this study is in line with theories of inhibition for impulse control (Duque and Ivry, 2009; Duque et al., 2010, 2014; Greenhouse et al., 2015). Accordingly, Fujiyama et al. (2012) did not find any evidence of MEP suppression in the elderly participants or any evidence of motor preparation.

Another procedure to motivate participants to prepare the response is the use of a reward. For instance, Freeman et al. (2014) used equiprobable Go and NoGo stimuli (square vs. triangle) presented in association with task-irrelevant cues, previously associated with a reward (green background) or no reward condition (purple background). Stimuli were interleaved by the presentation of a fixation cross for 3.5 seconds. Single TMS pulses were delivered 250 ms after stimulus onset and MEPs were recorded from task-relevant (FDI) and task-irrelevant (*abductor digiti* minimi, ADM) muscles. MEP amplitudes were normalized using data recorded for “Null” trials, that is, on trials in which, after the fixation cross, the word “Null” was displayed and subjects were required to remain at rest. For the FDI, they found an increase in CSE 250 ms following Go stimulus onset, in the presence of the previously rewarded stimulus, and, similarly, a suppression of MEPs at the same time point for successful NoGo trials, only when coupled with a motivationally salient stimulus. The authors reasoned that the use of equiprobable Go and NoGo stimuli, coupled with a non-salient (not previously rewarded) cue, failed to induce response preparation on such trials, therefore not requiring inhibitory mechanisms for impulse control. Salient cues instead always increased MEPs from the ADM, irrespectively of the presence of Go or NoGo stimuli, suggesting that salient stimuli induce a muscle-aspecific motor excitation that was later suppressed in the task-relevant muscle FDI on NoGo trials.

To better investigate whether reward-associated cues induce motor preparation, Freeman and Aron (2016) adopted equiprobable high- and low-reward predicting Go and NoGo stimuli. Specifically, while repetitive button presses in the presence of a Go stimulus would result in the reward predicted by the color-associated cue, no reward was ever delivered on NoGo trials. Nonetheless, the same colored cues were presented to participants, with the hypothesis that the simple presentation of a colored cue associated with a reward would be sufficient to induce motor preparation. Single TMS pulses were delivered at 100, 150, 200, and 250 ms following stimuli onset. MEPs were recorded from the task-relevant muscle FDI and normalized using baseline MEPs collected on some trials 500 ms before stimulus onset. The results showed a significant effect of reward type on behavioral and neurophysiological measures, and relevant to the present review, we will only discuss high reward trials. Go trials elicited a linear increase in motor excitability across the four time points, while on NoGo trials an initial MEP increase (starting 100 ms post-stimulus) was followed by a significant decrease (from 150 ms post-stimulus). This expected early activation late inhibition pattern was linked, according to the authors, to a reward-predicting cue-associated motor preparation, followed by inhibition on NoGo trials. While MEP suppression on NoGo trials was significantly different than baseline at the 250 ms post-stimulus time point, CSE levels on both Go and NoGo trials were not significantly different than pre-stimulus baseline levels at the first two time points. This pattern of results is reminiscent of the findings of Leocani et al. (2000), but whether the stimulus-induced activation up to 100 ms post-stimulus is muscle-aspecific cannot be ascertained in the present study, as Freeman and Aron (2016) only recorded MEPs from the task-relevant muscle FDI. In conclusion, the authors interestingly showed that (high) reward-predicting cues, coupled with Go stimuli, can also induce motor preparation on unrewarded NoGo trials. Future studies employing similar paradigms should consider that motor preparation follows stimulus onset, and as a consequence, inhibitory mechanisms on NoGo trials are delayed.

Saumur and Mochizuki (2018) adopted an acoustic version of the simple RT and the GNG tasks, although the frequency and durations of the tones used as WS and ISs are not specified. ISs followed the WS tone after a 3-s long foreperiod, and single pulse TMS was delivered 2 seconds after WS onset. MEPs were recorded from the right tibialis anterior, while participants were asked to dorsiflex the right foot in response to Go stimuli. Despite differences in the two tasks, the authors found MEPs to always be higher than baseline. Unfortunately, they only tested one stimulation time point during a long foreperiod, limiting the interpretation of these results.

In the attempt to isolate the relative contribution of distinct sets of excitatory inputs to corticospinal neurons during motor preparation and inhibition, Hannah et al. (2018) used single pulse TMS over M1 and changed the coil orientation to induce electrical current in a posterior-anterior (PA) vs. anterior-posterior (AP) direction. The authors reasoned that if inhibition for impulse control is responsible for the CSE suppression during motor preparation, then both types of stimulation should induce the same effect. Alternatively, if only some inputs are suppressed to increase the signal-to-noise ratio in the motor cortex (Hasbroucq et al., 1997; Touge et al., 1998; Duque and Ivry, 2009), the two stimulation paradigms will generate different results. Right and left FDI muscles were recorded while participants performed a set of cognitive tasks, including a Go/NoGo task, during which a WS (500 Hz tone for 150 ms) preceded Go (green visual stimulus) or NoGo (red visual stimulus) ISs, presented for 75 ms after a foreperiod of 2 seconds. Participants performed blocks of AP and PA TMS trials, and each block included Go and NoGo trials with a relative ratio of 3.3:1. TMS pulses could be delivered at different time points; however, they differed between Go and NoGo trials. TMS pulses on Go trials could be delivered at WS or IS onset, or at the estimated 35% or 70% or mean RT, while TMS pulses on NoGo trials were only delivered at these last two time points. Therefore, each time TMS was delivered at WS with the concomitant presentation of the WS tone, participants could predict the subsequent appearance of the Go stimulus and prepare the response (right index finger flexion) accordingly. The authors reported that MEPs in the task-relevant muscle recorded on Go trials when the TMS pulse was delivered at the end of the foreperiod (IS onset) were differentially modulated by AP and PA TMS. However, they were significantly reduced compared to catch trials, in which a TMS pulse was delivered at the expected time of WS onset (baseline). Since no TMS at WS or IS time points was delivered on NoGo trials, the authors normalized MEPs using data from the IS TMS trials (only Go trials), and the results show that a similar CSE suppression for both AP and PA TMS types occurs at the 70% RT time point. These effects suggest that, while reactive inhibition might entail a gating of all corticospinal output neurons, premovement CSE modulations involve a finer tuning of corticospinal neuron excitability.

## General Discussion

From all the studies discussed above, we can conclude that the Go/NoGo (GNG) task, despite its apparent simplicity, can engage different inhibitory mechanisms that may co-occur with motor preparation, or follow it, depending on the task requirements and participants’ behavior. Due to the nature of the task, the uncertainty regarding the identity of the upcoming stimulus (Go or NoGo) may engage proactive inhibition, a cognitive control mechanism thought to induce a cautious behavior resulting in two potential cognitive strategies: slow down responses to avoid errors (responses on NoGo trials) or exert an active motor suppression (Jaffard et al., 2007; Boulinguez et al., 2008, 2009; Duque and Ivry, 2009; Duque et al., 2010; Aron, 2011; Cai et al., 2011). Both NoGo probability and the potential presence (and duration) of foreperiods, on delayed GNG paradigms, can modulate the intensity of proactive inhibitory mechanisms. Furthermore, despite the GNG task requires a unimanual response on Go trials, inhibitory mechanisms for action selection may occur (Burle et al., 2004; Duque et al., 2010). Finally, the presentation of the NoGo signal might induce reactive action inhibition that can operate selectively on the task-relevant muscle, or globally, depending on how much the response was prepared in advance, and on time pressure (Aron and Verbruggen, 2008; Hannah et al., 2018). All these inhibitory mechanisms can modulate the excitability of the corticospinal tract, of which MEPs are a measure, at different time points during the trial (for a review, see Bestmann and Duque, 2016). The time of TMS pulses to record MEPs, the task structure (e.g., ratio of Go and NoGo stimuli, presence and duration of foreperiods), the inter-individual variability in impulsivity levels (Hoegl et al., 2012), and the excitability of the corticospinal tract (Greenhouse et al., 2017) are all factors that can potentially affect the results of TMS studies using the GNG task to investigate action inhibition. To control for this variability and to increase the reproducibility of GNG studies, we suggest that any experimental protocol to study action inhibition should always have a way to determine whether responses were prepared before the onset of imperative stimuli. To this aim, in the next section, we will compare the results of the previously reported studies to TMS studies that adopted other cognitive task (single and choice RT task) to comment on the possible cognitive control mechanisms involved in motor preparation.

## Motor Preparation in go and NoGo trials

The majority of single pulse TMS studies that adopted the GNG task to investigate action inhibition have reported an increase of CSE measured from the task-relevant muscle following Go stimuli, in line with classical findings of premovement facilitation (Tanji and Evarts, 1976; Rossini et al., 1988). Exceptions are represented by the studies of Fujiyama et al. (2012) and Hannah et al. (2018), in which the authors report Go trials-related initial preparatory suppression followed by motor facilitation. While at first their result might seem counterintuitive, it is actually more in line with current theories of inhibition for motor preparation. Cumulating evidence of preparatory suppression come from different types of choice and simple RT tasks, and while some authors argue that impulse control mechanisms aimed at preventing the anticipated execution of the prepared response are responsible for this effect (Touge et al., 1998; Duque and Ivry, 2009; Sinclair and Hammond, 2009), others support a less stringent hypothesis (subthreshold hypothesis), claiming that the excitability of corticospinal neurons is modulated to enhance the signal-to-noise ratio (Hasbroucq et al., 1997).

The results from NoGo trials are more variable, with most studies reporting global motor suppression starting from 100 ms post-IS, one study not reporting any changes in CSE (Fujiyama et al., 2011), while others finding motor facilitation, followed by inhibition (Leocani et al., 2000; Freeman and Aron, 2016). While differences in the adopted paradigms and recorded muscles could partially explain these different results, we suggest that a major source of variability derives from motor preparation. Fujiyama et al. (2012) found initial motor suppression (as in Go trials, probably due to motor preparation) that remained constant across the remaining time points, suggesting that the initial inhibitory control over the activated motor response was likely sufficient to prevent the execution of the planned action (Duque et al., 2010, 2014; Greenhouse et al., 2015). Interestingly, Fujiyama et al. (2012) adopted that rare NoGo stimuli (30% of the trials) and imperative signals (Go and NoGo) were preceded, 500 ms before, by a warning signal (WS). Under these experimental conditions, participants are more likely to prepare the response irrespectively of trial type, and the late response in the task-relevant muscle is facilitated, immediately before action execution (at ½ and ¾ of the PreMT). In fact, MEPs recorded at all time points during Go and NoGo trials were higher than resting levels. However, it remains unclear why the authors only found evidence for motor preparation (MEP suppression) *after* IS onset and not before (e.g., 250 ms following the WS). If participants prepared the response upon the presentation of the WS, they should have found suppressed MEPs also at this time point. We know from previous studies that 250 ms are sufficient to prepare the response (Bertelson, 1967; Touge et al., 1998; Tandonnet et al., 2010) and inhibition for preparation can be found as early as 100 ms into a 500 ms long foreperiod (Lebon et al., 2015).

A similar NoGo suppression was also reported by Freeman et al. (2014), using salient stimuli that were previously associated with a reward. However, both Leocani et al. (2000) and Freeman and Aron (2016) found initial facilitation, later followed by inhibition. In both studies, motor preparation is likely to occur at IS onset and not earlier. However, why would motor preparation not result in suppressed MEPs, as it occurred in Fujiyama et al. (2012) and many studies adopting delayed RT tasks? While in Freeman and Aron (2016) it is possible that the additional excitatory drive triggered by salient cues counteracts the presence of inhibitory influences to M1, the question remains for Leocani et al. (2000). One possibility is that the adoption of auditory stimuli, combined with unpredictable TMS pulses delivered at random times between 20 and 400 ms post-IS, induced fast M1 activation, without the concomitant involvement of inhibitory mechanisms, that occurred later. Several studies, in fact, suggest that, when participants have less time to prepare the response (such as during short foreperiods), time preparation speeds up M1 activation (Tandonnet et al., 2003, 2012; Davranche et al., 2007; see Vidal et al., 2018 for a review), whereas inhibitory mechanism may not be concurrently activated. We recently reported evidence of fast and muscle-selective CSE enhancement for short foreperiods (150 and 300 ms), without evidence of proactive inhibition, using a simple RT task (Ficarella and Battelli, 2018). While Leocani et al. (2000) did not adopt a delayed GNG task, the unpredictable TMS pulses might have alerted participants, inducing them to respond quickly on Go trials, engaging M1 in a task-aspecific manner, as confirmed by the bilateral facilitation found in the 20-100 ms premovement temporal window. Thus, late inhibitory mechanisms, activated upon the identification of NoGo stimuli, likely suppressed all responses to prevent errors.

## Conclusion

In this review, we compared the results of single pulse TMS studies adopting the GNG task, and we suggested that one major source of variability, which greatly limits data reproducibility, lies in motor preparation *before* the onset of Go and NoGo stimuli. Adopting mechanisms to motivate participants to prepare the response in advance, such as using rare NoGo stimuli, presenting a warning signal shortly before IS onset, and, to some extent, asking participants to weakly precontract the task-relevant muscles, generates results that are comparable to other studies on action inhibition (Duque and Ivry, 2009; Duque et al., 2010, 2014; Fujiyama et al., 2012; Greenhouse et al., 2015). Comparing MEPs to resting levels and normalizing them to a baseline (trial onset) is good practice to test whether participants prepared the response in advance.

To conclude, failures in the inhibition of prepotent responses can be due to deficits in the motor system reactivity to external stimuli or to a deficit in inhibitory control (DeYoung et al., 2011). This is not trivial because, to investigate and compare normal individuals with clinical populations, such as ADHD and Parkinson’s disease, it is paramount to control for motor preparation variability when disentangling motor preparation from action inhibition.

## Author Contributions

The present manuscript has been written by SF and revised by LB.

### Conflict of Interest Statement

The authors declare that the research was conducted in the absence of any commercial or financial relationships that could be construed as a potential conflict of interest.
